# Medically Relevant Assays with a Simple Smartphone and Tablet Based Fluorescence Detection System

**DOI:** 10.3390/s150511653

**Published:** 2015-05-20

**Authors:** Piotr Wargocki, Wei Deng, Ayad G. Anwer, Ewa M. Goldys

**Affiliations:** ARC Centre of Excellence in Nanoscale Biophotonics, Macquarie University, North Ryde 2109, NSW, Australia; E-Mails: piotr.wargocki@mq.edu.au (P.W.); wei.deng@mq.edu.au (W.D.); ayad.anwer@mq.edu.au (A.G.A.)

**Keywords:** smart phone, fluorescence, polarization, trypsin, collagenase, clinical assays

## Abstract

Cell phones and smart phones can be reconfigured as biomedical sensor devices but this requires specialized add-ons. In this paper we present a simple cell phone-based portable bioassay platform, which can be used with fluorescent assays in solution. The system consists of a tablet, a polarizer, a smart phone (camera) and a box that provides dark readout conditions. The assay in a well plate is placed on the tablet screen acting as an excitation source. A polarizer on top of the well plate separates excitation light from assay fluorescence emission enabling assay readout with a smartphone camera. The assay result is obtained by analysing the intensity of image pixels in an appropriate colour channel. With this device we carried out two assays, for collagenase and trypsin using fluorescein as the detected fluorophore. The results of collagenase assay with the lowest measured concentration of 3.75 µg/mL and 0.938 µg in total in the sample were comparable to those obtained by a microplate reader. The lowest measured amount of trypsin was 930 pg, which is comparable to the low detection limit of 400 pg for this assay obtained in a microplate reader. The device is sensitive enough to be used in point-of-care medical diagnostics of clinically relevant conditions, including arthritis, cystic fibrosis and acute pancreatitis.

## 1. Introduction

Fluorescent assays are widely used across the life sciences. Fluorescent assay readout can be carried out by a variety of devices. Most commonly, a microplate reader is used where a number of wells (frequently 96) filled with the assay solution can be examined at once [1,2]. In most cases, assay readout requires professional research laboratory equipment. There is a need to develop easy to use, portable and affordable systems for assay readout as these are preferred for point-of-care applications.

Trypsin and collagenase are clinically relevant biomarkers found in high concentration in many relevant diseases. Plasma levels of trypsin rise to almost 30 times the physiological level in patients with acute pancreatitis [3]. Elevated levels of trypsin are also associated with associated with cystic fibrosis and haemorrhagic shock [4,5]. Collagenase expression increases in rheumatoid arthritis and traumatic injuries [6] and its concentration can be used as a marker for the degree of synovial inflammation. The elevated levels of collagenase in chronic inflammation lead to the degradation of cellular matrix resulting in impaired joint function [7].

Commercial assays to detect collagenase and trypsin typically use a sandwich immunoassay approach with two antibodies to analyse the amount of collagenase or trypsin in samples. Radio-immunoassays have also been used to detect serum trypsin level [8,9], however, fluorescent reporters are much safer and more convenient to use especially in point-of-care situations. In this study we used commercial assays for trypsin and collagenase with a very common fluorescein isothiocyanate (FITC) dye as a reporter. This dye has excellent fluorescence quantum yield, good water solubility, and it reacts with amino groups of most proteins. It is commonly applied in a variety of assays, including for FITC-casein [10], fibrinogen [11,12], Ca^2+^-ATPase [13], NO [14], C-reactive protein [15], and many other biomolecules.

Consumer electronics, such as cell phones, smartphones, and flatbed scanners, are widely recognised for their wide-ranging capacity to be reconfigured for a variety of purposes, such as advanced positioning, metal detection, measurement of vital functions, and, more recently, fluorimetry and biochemical analysis [16,17]. Ozcan and his team pioneered the use of smartphone-based devices for biomedical applications, including imaging water-borne pathogens and viruses [18,19], dark field imaging of these pathogens and white-blood cells [20] and blood testing devices [21]; some of these have been recently reviewed in [22]. Other authors have been developing systems for fluorescent detection of human cells and pathogens, as well as molecular analytes, such as proteins and nucleic acids [23]. A smartphone-based device with a microfluidics accessory has been recently applied for rapid immunoassay diagnostics in genuine point-of-care settings [24]. Multiplex detection of pathogen DNA, extracted from patient samples, following an amplification step, has also been demonstrated [25]. All of them, however, are based on an additional unit that is attached to the phone, usually with specialised optics and excitation sources [18,19,20,21,26,27].

In this paper, we describe a cell phone-based portable bioassay platform with a minimal number of commonly available components, and its application to selected medically relevant fluorescent assays. The system uses a screen from a commercial tablet as an excitation light source, linear polarisers that separate the excitation light from the fluorescence signal being readout, a smartphone as a camera that takes still pictures of an assay well, and a black box protecting the assay against ambient light. Specialised software is not required and setting up the standard commercial tablet and smart phone is very straightforward. The assay readout is simply carried out by taking an image of the assay well. After image acquisition, the pictures are post processed to quantify the result. The picture is cropped to select the assay area, an appropriate RGB colour channel is selected, in this case, green, matching the characteristics of the assay reporter dye, fluorescein. Finally, adaptive filtering [28] is applied to the assay image and the mean pixel intensity is calculated. In order to assess system’s performance and sensitivity, two kinds of assays for the detection of trypsin and collagenase were performed of this smart phone-based device, and on a commercial fluorimeter, as a benchmark.

## 2. Experimental Section

**Figure 1:**
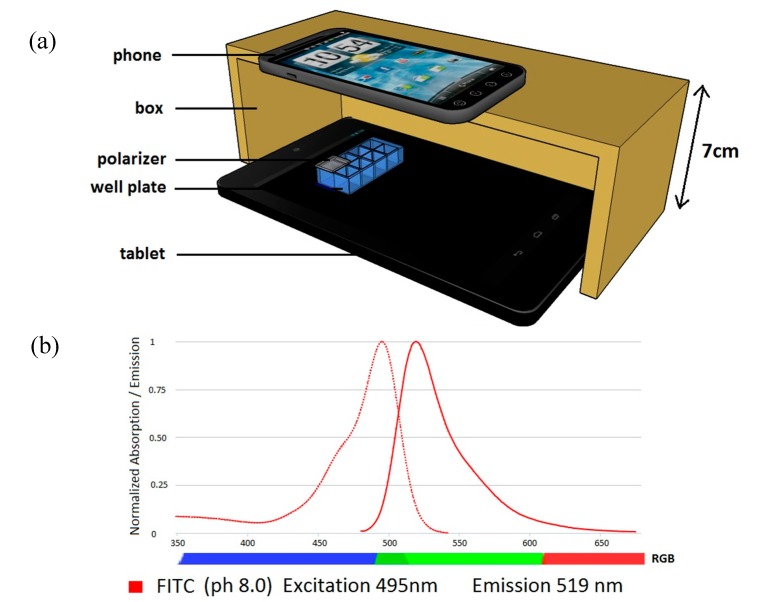
(**a**) Schematic diagram of the device cross-section; (**b**) Excitation and emission spectra of fluorescein in comparison to a typical RGB colour range.

We first present the overview of our hardware setup. The device used to perform the measurements was constructed as shown in Figure 1a. The well slide with assay samples were placed on the surface of the tablet screen. The excitation source was a bright single square, 13 mm in size (165 pixels), generated on an otherwise black tablet screen. This square was blue with an RGB value of (0;0;255) matching the excitation spectrum of fluorescein shown in Figure 1b, where we also display typical spectra of RGB colours. The square was slightly larger than the assay well and it was entirely covered with a polarizer. The tablet screen used here emitted linearly polarised light and the polariser was aligned perpendicularly in relation to tablet screen polarization. The tablet, assay well, and polariser were placed inside of a box with an opening for the camera; this box shielded the detection system from ambient light. The phone was placed on the top, to be able to capture images from an appropriate distance (in our case 7 cm).

The tablet used in our device was an Asus Nexus 7 (2013) with full HD (1200 × 1920) resolution and an IPS-LCD screen. Its brightness was set to 100%, producing 583 cd/m^2^ for white colour [29]. The tablet displaying blue colour was examined separately using a spectrophotometer, which confirmed light emission within the 400–500 nm range overlapping with the fluorescein excitation spectrum.

We now discuss the required characteristics of the cell phone. In this work, the HTC EVO 3D (X515) cell phone was used to capture assay images with its built-in camera. In order for these images to be in-focus, the height of the box needed to be adjusted, but also the camera settings needed to be controlled by the user to a sufficient degree. In particular, the settings for the ISO value and the white balance needed to be chosen manually. To determine the best white balance option, an experiment was conducted, where a green square (0;255;0 in the RGB colour model) was displayed on the tablet screen and images were taken with different settings. The best combination of settings was chosen to be the one with the highest green pixel value and minimal red and green pixel values. The optimised phone camera and tablet settings are listed in Table 1.

**Table 1:** Tablet and phone settings.

Phone Camera Settings		Tablet Settings	
ISO	800	Square side	165 px (13 mm)
White balance	daylight	Square colour	255 Green
Image resolution	5 Mpx	Brightness	100%
Flash	off		
Automatic correction	off		
Automatic sharpening	off		

A polariser is an essential part of our device. The polarizer used for this work was a Moxtek PFU04C wire grid polarizer, with size 12.5 mm × 12.5 mm. This wire grid polariser is characterised by high contrast, large acceptance angle, and broadband performance. Due to this construction it has excellent durability and long lifetime, and its crossed transmittance is low (0% ± 0.5%). The polarizer was placed on the top of the well with the sample, to prevent blue excitation light from interfering with the readout carried out in the green channel. Since the tablet screen is already polarized, only one polarizer was used in this instance. In order to verify the effectiveness of this polariser in blocking the excitation light, the following experiment was conducted: The blue square on the tablet screen was covered with the polarizer at 90 degrees, relative to the direction of screen polarisation. Further, an image was taken and the signal in the green readout channel was measured. In this case, we registered the mean green pixel value of 7, which was comparable to the background noise value of 4. The tablet transmittance thus obtained was less than 2%, which means that the polariser was indeed effective to block excitation light.

**Figure 2:**
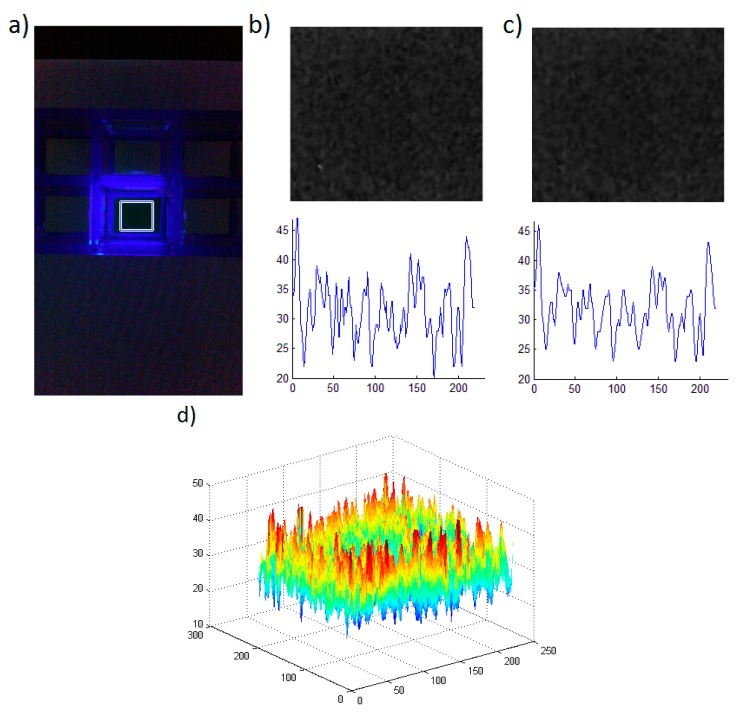
Image processing steps. (**a**) Initial image with the AOA rectangle highlighted; (**b**) Green channel image (in grayscale) and a representative cross-section of screen intensity presenting noise amplitude; (**c**) Image filtered with adaptive Wiener filter and a representative cross-section of intensity presenting noise amplitude; (**d**) Pixel intensity map of the AOA after adaptive filtering.

Accurate quantification of the assay signal, especially at low analyte levels, requires image processing, which was performed in the following way: The area of analysis (AOA) was determined from the original image. The AOA used here was a 200 px × 220 px rectangle, cropped from the signal part of the image (Figure 2a). Subsequently, the resulting image was split into 3 colour channels––red, green and blue. Since the signal emission for fluorescein is green, and it should not contain any blue colour, the green channel is selected for further analysis (see Figure 2b) where the intensity of green channel is shown as a grayscale image). In the next step, adaptive filtering [28] was used to improve image quality and reduce noise (Figure 2c). The Wiener filter used here estimates the local mean and variance around each pixel, as follows:

(1) $$\mu =\frac{1}{NM}\sum_{n_{1},n_{2}\in \eta }a\left(n_{1},n_{2}\right)$$

and:

(2) $$\sigma^{2}=\frac{1}{NM}\sum_{n_{1},n_{2}\in \eta }a^{2}\left(n_{1},n_{2}\right)-\mu^{2}$$

where η is the *N*-by-*M* local neighbourhood of each pixel in the image A. The Wiener filter then creates pixelwise estimates:

(3) $$b\left(n_{1},n_{2}\right)=\mu +\frac{\sigma^{2}-v^{2}}{\sigma^{2}}\left(a\left(n_{1},n_{2}\right)-\mu \right)$$

where $v^{2}$ is the noise variance. If the noise variance is not given, the Wiener filter uses the average of all the local estimated variances. Figure 2d shows a typical AOA pixel intensity after Wiener filtering. The results were considered reliable when approximately uniform intensity distribution across the whole AOA was observed without noticeable peaks.

The assay readings were produced in the following way. As indicated earlier, green pixel values were used in these calculations. The average pixel intensity and standard deviation values were calculated from the assay AOA after adaptive filtering. We then established the background by imaging the assay well, filled with buffer only, without any fluorescent substances. The background was calculated from the respective AOA, also after adaptive filtering. The assay signal is the average pixel intensity of the assay AOA minus the average pixel intensity of the background AOA. The SNR was calculated for each measurement as an average pixel intensity of the filtered AOA with subtracted background noise and divided by the standard deviation of pixel values in the filtered AOA. The low detection limit was considered to be achieved when the SNR signal to noise ratio (SNR) had the value of 3.

The samples for the analysis were prepared as follows. We used the Pierce fluorescent protease assay kit (catalogue #23266, Thermo Scientific, Inc. Hudson, NH, USA) and type 1 collagenase assay kit (B-Bridge International, Inc., Cupertino, CA, USA, catalogue #AK07) and collagenase from *Clostridium histolyticum* (Sigma-Aldrich, St. Louis, MO, USA, Sigma Prod. No. C0130). For the trypsin assay, trypsin solution with different concentrations was mixed with FTC-Casein solution, followed by incubation at room temperature for 60 min. For the collagenase assay, collagenase enzyme crude from *Clostridium histolyticum* (Sigma Aldrich) was prepared at different concentrations (60, 40, 20, 10, 5, 2.5, 1.25) µg/mL in TESCA buffer (50 mM TES, 0.36 mM calcium chloride, pH 7.4 at 37 °C). The type 1 collagenase assay kit (B-Bridge international, Inc. USA) was used to detect collagenase concentration in prepared solutions.

In order to prepare the substrate solution for the enzymatic reaction, 1 mL of fluorescent-labelled collagen and buffer A (supplied in collagenase kit) were mixed and kept on ice until needed. One hundred microlitres of substrate solution and collagenase enzyme solutions with different concentrations were added into a microtube and mixed thoroughly. Samples were then incubated at 35 °C for 2 h. Six hundred microlitres of cooled buffer B (supplied with the collagenase kit) was added to each tube and kept in ice for 15 min. All tubes were centrifuged at 10,000 rpm for 10 min. The fluorescence was measured from the supernatant. We emphasise that the fluorescent reporter used in both examined assays was fluorescein, as indicated previously, with an excitation maximum at 494 nm and emission maximum at 521 nm.

**Figure 3:**
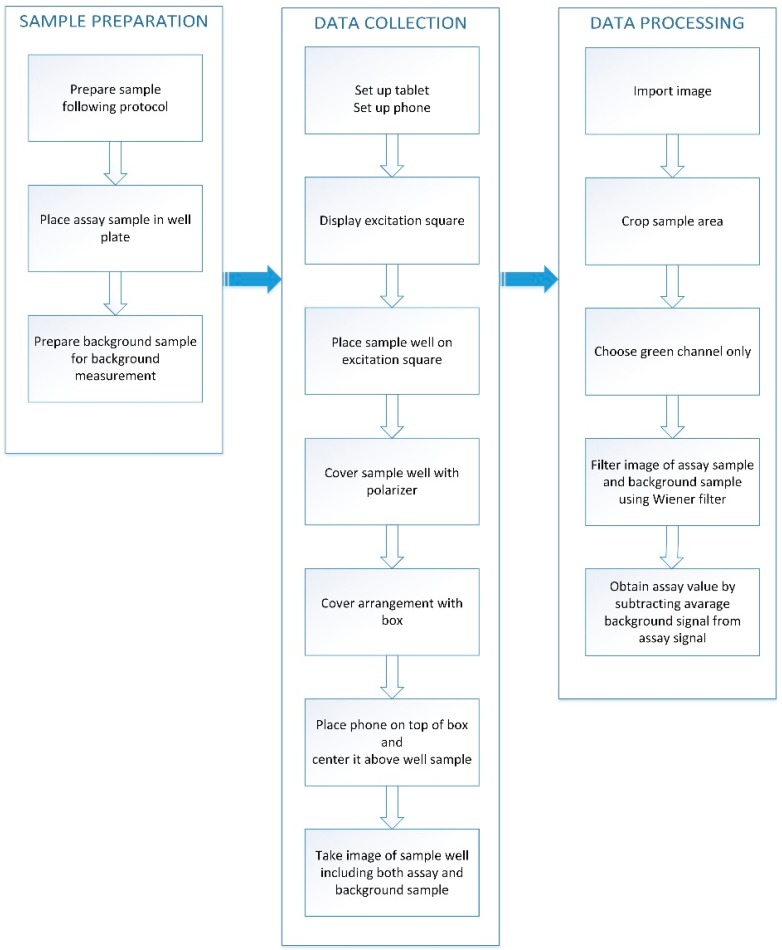
The assay workflow.

In order to perform measurements using the smart phone system, solutions of trypsin and collagenase assays were added into 8 well chamber slides (BD Biosciences). Each well was a cube with a 9-mm size. Two hundred and fifty microlitres of solution were placed in each well and this yielded 1.4 mm of the sample height. The assay workflow is illustrated in Figure 3. Each sample was imaged 3 times and each photograph was processed 3 times using a different AOA each time. These measurements that were used to calculate the final mean value for the sample and its uncertainty. For comparison, fluorescence emission from the same solutions were also measured at the excitation wavelength of 480 nm by using a CARY Eclipse fluorimeter (Agilent Technologies, Santa Clara, CA, USA).

## 3. Results

**Figure 4:**
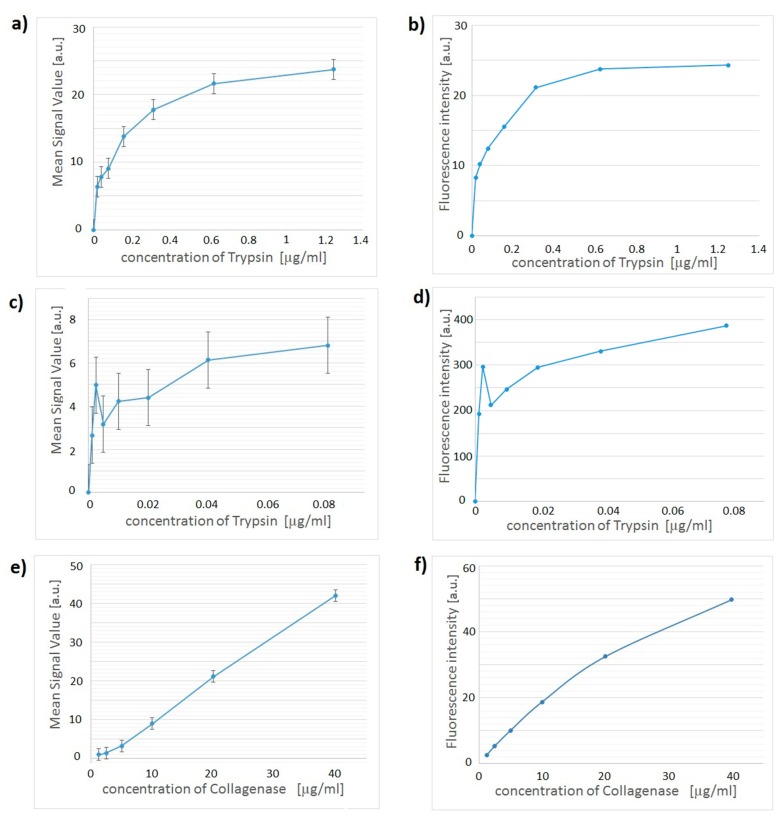
Assay signal as a function of analyte concentration for trypsin and collagenase assays. (**a**) Trypsin assay with smartphone device; (**b**) Trypsin assay with Cary Eclipse readout with photomultiplier detector voltage option set to ‘Low’. Uncertainty of each data point is 0.1; (**c**) Trypsin assay with smartphone device at low concentrations; (**d**) Trypsin assay at low concentrations with Cary Eclipse readout with photomultiplier detector voltage option set to ‘Medium’. Uncertainty of each data point is 2.7; (**e**) Collagenase assay with smartphone device; (**f**) Collagenase assay with Cary Eclipse readout with photomultiplier detector voltage option set to ‘Low’. Uncertainty of each data point is 1.1.

Several sets of samples were carefully prepared and examined using the device described here, and with a specialized fluorimeter. The results for trypsin and collagenase are presented in Figure 4. Figure 4a shows the results of trypsin assay measured on our smartphone system, while Figure 4b shows the same trypsin assay samples examined by standard fluorimetry. Another sample set with lower concentrations of trypsin was examined in order to determine the low detection limit; these results are presented in Figure 4c. Error bars in this figure are the same as in Figure 4a, but they appear larger than in Figure 4a due to a different scale on the vertical axis. Figure 4d shows the results for the same low trypsin density sample characterised by standard fluorimetry.

## 4. Discussion

Figure 4c presents results obtained using our smartphone device for low trypsin concentrations. From these data we obtain that the lowest measured concentration is below 3.72 ng/mL and the total quantity of detected trypsin was 930 pg within a 250 µL sample. This value is comparable to the low detection limit of 400 pg for the commercial assay obtained in a microplate reader. Thus, the presented device has been able to measure low concentrations of trypsin in combination with fluorescein as a fluorescent reporter.

The examination of trypsin level in blood is a standard procedure for newborns, because increased trypsin level may indicate cystic fibrosis [30,31]. Typical level of trypsin for a healthy child below one year of age is below 200 µg/L [30], while a trypsin level in a range of 200–1000 µg/L is an indication to carry out additional tests. The detection range of interest for cystic fibrosis application is, therefore, between 200 ng/mL and 1800 ng/mL [30]. With our low detection limit of 3.72 ng/mL the smartphone device is over 50 times more sensitive than what is required for the detection of cystic fibrosis.

By using our device we have also been able to achieve the low detection limit for collagenase of 3.75 µg/mL, which is 0.9375 µg in total in sample. The clinically relevant concentration range of collagenase in synovial fluid is between 1.6–11.7 µg/mL in patients with rheumatoid arthritis and patients with different grades of joint inflammation [32,33], hence, our measured concentration of 3.75 µg/mL is within the range of clinically relevant values.

We emphasise that the device presented here is largely technology platform-independent, making it suitable for a range of resource-poor settings. Alternative cell phone models with a camera can also be used in this device, as long as that camera can be prevented from making automatic adjustments of the relevant settings. For example Apple iPhones and Android devices (e.g., Samsung Galaxy series) are suitable replacements. Other types of mobile devices can also be used in this application as the light source, for example Apple iPads, Samsung Galaxy Tab family, or even various models of cell phones. The key parameters determining their suitability are screen brightness, colour projection (gamut) and polarization of the screen. Many commercial devices are compared in relation to these parameters on specialized Internet websites [29,34]. In alternative devices with different screen resolution or screen size, the size of the excitation square should be adjusted. The screens in some mobile devices (such as iPads) produce light polarized at a 45-degree angle, so the polarizer needs to be set properly for the excitation light to be fully extinguished. If the screen is not polarized, or insufficiently polarized, then two polarizers should be used.

We also emphasise that the system described here will tolerate various modifications (e.g., excitation light wavelength, readout channel) without affecting core functionality, and it can be can adapted to become universally applicable fluorescence detection device. Due to this flexibility, the device will also be able to be used with alternative fluorescent assays and other fluorophores.

With increasing availability and wide adoption of smartphone technology, including in the developing world, come increasing opportunities for their application in biomedical diagnostics, with multiple authors reporting being able to use smartphone-based systems for medically relevant assays [16,17,24,25,35]. However, in many cases, this requires fairly complex technologies in addition to a phone. One of the limitations is the sample preparation procedure required for specific assays, which usually is managed by a specialised add-on “dongle” [17,24] of which complexity depends on the assay reporting scheme. For example, in Reference [24], a silver enhancement step, following binding of gold-labelled antibodies, required complex fluidics. The complexity of fluidics grows with multiplex requirements, especially if an analyte amplification step is required [17]. The readout itself is typically carried out by a combination of standard optical components, such as excitation lasers, filters, and lenses [25], or optical filters and optical gratings [16] with the phone camera only performing the function of a detection device. References [16,35] exploit aspects of imaging, and Reference [35] additionally uses screen illumination for colorimetric readout and RGB channels for limited multiplexing. In this context, the presented system for fluorescent assays sits at the lower end of the scale for complexity and cost. It uses the RGB channel split and a polariser instead of optical filters and a screen as the light source. Hence, in the present system, all required system engineering is contained in its software, which is freely available at no cost. A graphic-user interface driven application for our system with all the required functionality to carry out assays can be downloaded from the website www.cnbp.org/smartphone_biosensing.

## References

[1] Lorenzen A., Kennedy S.W. (1993). A fluorescence-based protein assay for use with a microplate reader. Anal. Biochem..

[2] Wang H., Joseph J.A. (1999). Quantifying cellular oxidative stress by dichlorofluorescein assay using microplate reader1. Free Radic. Biol. Med..

[3] Mero M., Schröder T., Tenhunen R., Lempinen M. (1982). Serum phospholipase A2, immunoreactive trypsin, and trypsin inhibitors during human acute pancreatitis. Scand. J. Gastroenterol..

[4] Altshuler A.E., Penn A.H., Yang J.A., Kim G.-R., Schmid-Schönbein G.W. (2012). Protease activity increases in plasma, peritoneal fluid, and vital organs after hemorrhagic shock in rats. PLoS ONE.

[5] Heeley A.F., Watson D. (1983). Cystic fibrosis––Its biochemical detection. Clin. Chem..

[6] Walakovits L.A., Moore V.L., Bhardwaj N., Gallick G.S., Lark M.W. (1992). Detection of stromelysin and collagenase in synovial fluid from patients with rheumatoid arthritis and posttraumatic knee injury. Arthritis Rheum..

[7] Montrull H.L., Brizuela N.Y., Demurtas S.L., Strusberg A.M., Spitale L.S., Meirovich C.I. (2000). Collagenase production increases in rheumatoid arthritis and osteoarthritis synoviocytes incubated. Rev. Fac. Cien. Med. Univ. Nac. Cordoba.

[8] Lequin R.M. (2005). Enzyme Immunoassay (EIA)/Enzyme-Linked Immunosorbent Assay (ELISA). Clin. Chem..

[9] Artigas J.M., Garcia M.E., Faure M.R., Gimeno A.M. (1981). Serum trypsin levels in acute pancreatic and non-pancreatic abdominal conditions. Postgrad. Med. J..

[10] Spencer R.D., Toledo F.B., Williams B.T., Yoss N.L. (1973). Design, construction, and two applications for an automated flow—cell polarization fluorometer with digital read out: Enzyme-inhibitor (antitrypsin) assay and antigen—antibody (insulin—insulin antiserum) assay. Clin. Chem..

[11] Kinoshita K., Maeda H., Hinuma Y. (1980). Fluorescence polarization assay of plasmin, plasminogen, and plasminogen activator. Anal. Biochem..

[12] Pappenhagen A.R., Koppel J.L., Olwin J.H. (1962). Use of fluorescein-labeled fibrin for the determination of fibrinolytic activity. J. Lab. Clin. Med..

[13] Pick U., Karlish S.J. (1980). Indications for an oligomeric structure and for conformational changes in sarcoplasmic reticulum Ca^2+^-ATPase labelled selectively with fluorescein. Biochim. Biophys. Acta.

[14] Suzuki N., Kojima H., Urano Y., Kikuchi K., Hirata Y., Nagano T. (2002). Orthogonality of calcium concentration and ability of 4,5-diaminofluorescein to detect no. J. Biol. Chem..

[15] Devaraj S., Clos T.W.D., Jialal I. (2005). Binding and internalization of C-reactive protein by Fcgamma receptors on human aortic endothelial cells mediates biological effects. Arterioscler. Thromb. Vasc. Biol..

[16] Yu H., Tan Y., Cunningham B.T. (2014). Smartphone fluorescence spectroscopy. Anal. Chem..

[17] Edwards A.D., Reis N.M., Slater N.K.H., Mackley M.R. (2011). A simple device for multiplex ELISA made from melt-extruded plastic microcapillary film. Lab Chip.

[18] Wei Q., Qi H., Luo W., Tseng D., Ki S.J., Wan Z., Göröcs Z., Bentolila L.A., Wu T.-T., Sun R. (2013). Fluorescent imaging of single nanoparticles and viruses on a smart phone. ACS Nano.

[19] Koydemir H.C., Gorocs Z., Tseng D., Cortazar B., Feng S., Chan R.Y.L., Burbano J., McLeod E., Ozcan A. (2015). Rapid imaging, detection and quantification of Giardia lamblia cysts using mobile-phone based fluorescent microscopy and machine learning. Lab Chip.

[20] Zhu H., Yaglidere O., Su T.-W., Tseng D., Ozcan A. (2011). Cost-effective and compact wide-field fluorescent imaging on a cell-phone. Lab Chip.

[21] Zhu H., Mavandadi S., Coskun A.F., Yaglidere O., Ozcan A. (2011). Optofluidic fluorescent imaging cytometry on a cell phone. Anal. Chem..

[22] Zhu H., Isikman S.O., Mudanyali O., Greenbaum A., Ozcan A. (2013). Optical imaging techniques for point-of-care diagnostics. Lab Chip.

[23] Liu X., Lin T.-Y., Lillehoj P.B. (2014). Smartphones for cell and biomolecular detection. Ann. Biomed. Eng..

[24] Laksanasopin T., Guo T.W., Nayak S., Sridhara A.A., Xie S., Olowookere O.O., Cadinu P., Meng F., Chee N.H., Kim J. (2015). A smartphone dongle for diagnosis of infectious diseases at the point of care. Sci. Transl. Med..

[25] Ming K., Kim J., Biondi M.J., Syed A., Chen K., Lam A., Ostrowski M., Rebbapragada A., Feld J.J., Chan W.C.W. (2015). Integrated quantum dot barcode smartphone optical device for wireless multiplexed diagnosis of infected patients. ACS Nano.

[26] Navruz I., Coskun A.F., Wong J., Mohammad S., Tseng D., Nagi R., Phillips S., Ozcan A. (2013). Smart-phone based computational microscopy using multi-frame contact imaging on a fiber-optic array. Lab Chip.

[27] Mudanyali O., Dimitrov S., Sikora U., Padmanabhan S., Navruz I., Ozcan A. (2012). Integrated rapid-diagnostic-test reader platform on a cellphone. Lab Chip.

[28] Lim J.S. (1989). Two-Dimensional Signal and Image Processing.

[29] Nexus 7 (2013)––Mini Review. http://www.anandtech.com/show/7176/nexus-7-2013-mini-review.

[30] Crossle J., Elliot R.B., Smith P. (1979). Dried-blood spot screening for cytic fibrosis in the newborn. Lancet.

[31] Heeley A.F., Heeley M.E., King D.N., Kuzemko J.A., Walsh M.P. (1982). Screening for cystic fibrosis by dried blood spot trypsin assay. Arch. Dis Child..

[32] Maeda S., Sawai T., Uzuki M., Takahashi Y., Omoto H., Seki M., Sakurai M. (1995). Determination of interstitial collagenase (MMP-1) in patients with rheumatoid arthritis. Ann. Rheum. Dis..

[33] Clark I.M., Powell L.K., Ramsey S., Hazleman B.L., Cawston T.E. (1993). The measurement of collagenase, tissue inhibitor of metalloproteinases (TIMP), and collagenase-TIMP complex in synovial fluids from patients with osteoarthritis and rheumatoid arthritis. Arthritis Rheum..

[34] Mini Tablet Display Technology Shoot-out. http://www.displaymate.com/Tablet_ShootOut_4.htm.

[35] Vashist S.K., van Oordt T., Schneider E.M., Zengerle R., von Stetten F., Luong J.H.T. (2015). A smartphone-based colorimetric reader for bioanalytical applications using the screen-based bottom illumination provided by gadgets. Biosens. Bioelectron..

